# Back to the Middle Ages: Entomological and Botanical Elements Reveal New Aspects of the Burial of Saint Davino of Armenia

**DOI:** 10.3390/insects13121113

**Published:** 2022-12-01

**Authors:** Augusto Loni, Stefano Vanin, Antonio Fornaciari, Paolo Emilio Tomei, Valentina Giuffra, Giovanni Benelli

**Affiliations:** 1 Department of Agriculture, Food and Environment, University of Pisa, Via del Borghetto 80, 56124 Pisa, Italy; 2 Department of Earth, Environment and Life Sciences (DISTAV), University of Genova, Corso Europa, 26, 16132 Genova, Italy; 3 National Research Council, Institute for the Study of Anthropic Impact and Sustainability in the Marine Environment (CNR-IAS), 16128 Genova, Italy; 4 Division of Paleopathology, Department of Translational Research and New Technologies in Medicine and Surgery, University of Pisa, Via Roma 57, 56126 Pisa, Italy; 5 Accademia Lucchese di Scienze, Lettere e Arti, Via V. Veneto, 1, 55100 Lucca, Italy

**Keywords:** middle ages, Italian mummies, funerary archeoentomology, Tuscany, *Muscina*, Diptera, Coleoptera, Tineidae, Ichneumonidae, Julida, Scorpiones

## Abstract

**Simple Summary:**

In this research, we performed an entomological and botanical investigation to evaluate some historical records on the post-mortem history of Saint Davino Armeno (11th century). We formulated some intriguing hypotheses related to a potential exposure of the body after death, the presence of a wooden coffin, and the type of environment of his first burial. Arthropod data allowed us to state that Saint Davino was first buried into the soil, likely in a wooden coffin. Almost all sampled arthropods belong to species that usually colonize corpses in concealed conditions during later colonization waves. An exception was represented by *Muscina* (Diptera: Muscidae) species, which colonizes bodies during the first phases of decomposition. Notably, the phenology of the *Muscina* spp.—mainly active in late spring and early summer—supports the available information on the Saint’s death, which the hagiographic tradition places in early June. Lastly, botanical insights outlined that a significant number of herbaceous crops and trees were present in Tuscan medieval urban environments.

**Abstract:**

The natural mummy of Saint Davino Armeno (11th century) is preserved in the church of Saint Michele in Foro in the city of Lucca (Tuscany, Central Italy). The body of Davino is one of the oldest Italian mummies of a Saint, and his paleopathological study was performed in 2018. In the present research, we investigated the arthropod fragments and botanical remains collected from the body, coffin, and fabrics of Saint Davino. Entomological analyses outlined the presence of 192 arthropod fragments. Among these, Diptera, Muscidae (*Hydrotaea capensis* and *Muscina* sp.), and Phoridae (*Conicera* sp.) puparia were the most abundant. Regarding Coleoptera, Ptinidae (*Anobium punctatum*) were the most frequent, followed by Cleridae (*Necrobia* sp.), Trogidae (*Trox scaber*), Curculionidae (*Sitophilus granarius*), and Histeridae (*Gnathoncus*). Cocoons of Tineidae and Pyralidae moths were found, along with a propodeum joined to the petiole and a mesopleuron of an Ichneumoninae parasitoid. Numerous metamera of Julida and three scorpion fragments were also found. Botanical samples indicated the presence of a quite broad botanical community, including gramineous species, olives, evergreen oaks, and grapevine. Overall, entomological data allow us to argue that Saint Davino was first buried into the soil, probably in a wooden coffin, thus supporting the historical-hagiographic tradition according to which he was buried sub divo in the cemetery of Saint Michele. The preservation of the body as a natural mummy may have been facilitated by burial in a coffin that prevented direct contact of the corpse with the earth. Botanical remains offer confirmation of a late medieval urban environment rich in horticultural areas and trees, giving us a landscape that is very different from the current Tuscan city.

## 1. Introduction

“Mummies of Saints” are a particular category of Italian mummies [1], and they have been the object of several studies seeking to verify their authenticity and the state of their preservation [1,2,3,4]. They have historical and cultural relevance because the possession/ownership of the body of a saint was considered a symbol of power and identity in Christianity. The scientific study of the mummies of Saints is also essential to establishing the mummification process [5], which could has been natural, artificial, or facilitated [6,7,8], to reconstruct their history and the post-mortem events such as their transfer from primary to secondary burials. Although the mummification/dehydration process reduces the number of organisms (e.g., bacteria and insects) able to colonize a body [9,10], their presence can reveal important information in the reconstruction of the peri- and post-mortem events [3,11]. The study of the insects associated with human or other animal bodies of archaeological interest is the topic of funerary archaeoentomology. This discipline, coded in 1996 by the French entomologist Jean-Bernard Huchet [12,13] and widely applied in Italy [3,6,11,14], focuses on insects and other arthropods associated with human and animal cadavers from archaeological contexts. Funerary archaeoentomology provides useful information about the body taphonomy and the peri- and post-mortem events (e.g., displacements, places of conservation, and treatments) of the body over the centuries but also on potential contaminations of the bodies during their storage in collections and museums [2,6,15,16]. In addition, the analysis of botanical elements such as pollen [17,18], seeds, leaves, and fragments of wood is well established in archaeological investigation and provides a wide spectrum of information regarding the lifestyle, population’s migration, development of agricultural practices, food preparation, and funerary practices in past contexts, among others [5,19,20].

The mummified body of Saint Davino Armeno (?-†1050), one of the oldest known natural mummies of a saint in Italy, is preserved under the high altar in the church of Saint Michele in Foro in the city of Lucca (Tuscany, Central Italy). In the Middle Ages, Lucca was an important city along the Via Francigena, a major trade and pilgrimage route connecting France and Northern Europe with Rome. The hagiographic sources report that Saint Davino left Armenia and, after a long pilgrimage to Jerusalem and Rome, arrived in Lucca in the year 1050. He died in the Tuscan city during a stop of the pilgrimage to Santiago de Compostela. The veneration of his body is already attested in the 12th century, probably amplified by its fine preservation [21]. From historical sources, including the oldest handwritten source on the life of the saint dating back to the end of the 12th century (*Tractatus de vita et obitu atque miraculis Beati Davini confessoris*, Archives of the Vicariate of the Chapter of San Giovanni in Laterano, ms. A. 79, ff. 280e–281v) [22], it is known that he had a first burial in the cemetery outside the church of Saint Michele, but after some miraculous events that occurred on his tomb, such as the growth of a vine (*Vitis vinifera* L.) plant with fruits capable of curing any disease, the body was exhumed, found miraculously intact, and transferred inside the church of Saint Michele, one of the most important churches of the city of Lucca [23]. It was initially (11th century) preserved in a terracotta sarcophagus, which in the 12th–13th centuries was placed in a monumental marble tomb. In 1592, the body was placed under the high altar of the church, and in 1646 the medieval sarcophagus was replaced by a cypress wood case exposed to the veneration of the faithful during the celebrations in his honour which take place every year on June 3rd [23].

In the present study, an entomological and botanical investigation was performed to verify some historical records about his post-mortem history. The main hypotheses of this research were related to a potential exposure of the body after death, the presence of a wood coffin, and the type of environment of his first burial.

## 2. Materials and Methods

### 2.1. Historical Context and Sampling

**Figure 1:**
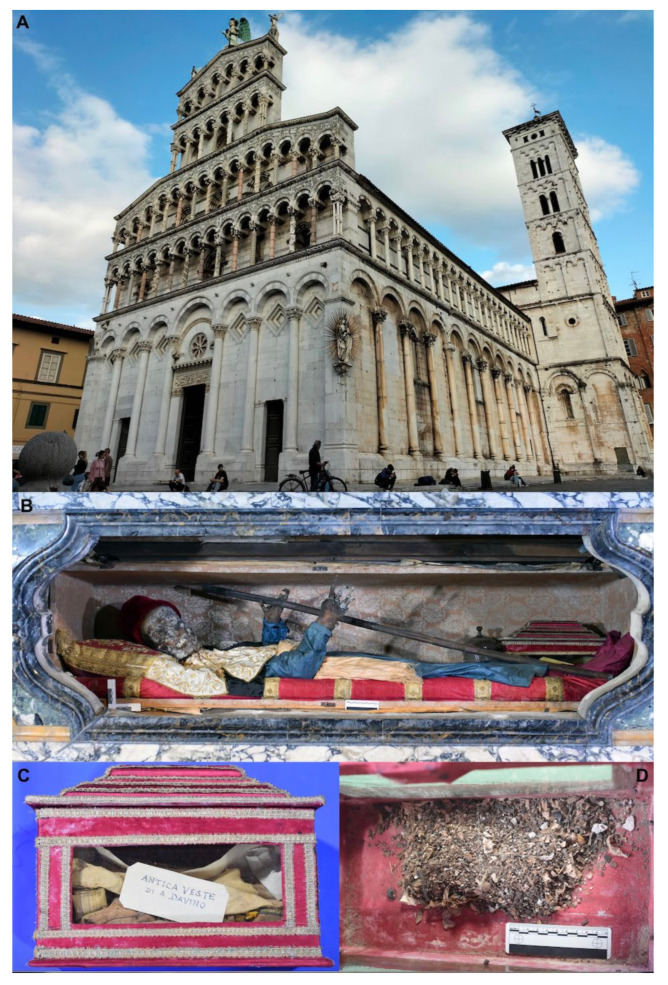
(**A**) External view of the magnificent church of Saint Michele in Foro (Lucca, Central Italy), where the body of Saint Davino Armeno is preserved (**B**). Whole view (**C**) and internal detail (**D**) of the small box kept in the Saint Davino reliquary, containing the remains of ancient fabrics and material made up of powders collected during the ancient canonical reconnaissance of the body, which were done in 1547, 1592, 1646, and 1669.

The analysis of Saint Davino’s remains published by Fornaciari et al. [24,25] reported a partially skeletonized natural mummy of a man of about 25 years, 1.70 m tall. The palaeopathological study revealed two traumatic lesions of the skull with long-term survival: a superficial sharp force lesion on the left frontal bone, perhaps caused by a toothed blade, and an elliptical wound produced by a blunt weapon on the right coronal suture, with evident signs of surgical treatment [25]. Probably because of these lesions on the head, Saint Davino is traditionally invoked against headaches. In March 2018, the mummy was transferred from the high altar to a temporary lab set up in a transept of the church of Saint Michele (Figure 1) under the same conditions of temperature and humidity.

The study of the mummy, performed by the Division of Paleopathology of the University of Pisa, included undressing, macroscopic examination, endoscopy of the oral cavity, and CT of the total body. Very small tissue samples were taken for further study. The body, in balance with the environmental conditions of the church, was then redressed and replaced under the high altar. During the undressing and study of the body, various samples containing remains of entomofauna and different botanical elements were recovered. Together with the body, a small box was kept in the reliquary of the saint containing the remains of ancient fabrics and material made up of powders collected during the ancient canonical reconnaissance of the sacred body (Figure 1), which took place in the years 1547, 1592, 1646, and 1669 [23]. All these materials were sampled integrally and analysed for the present study. Arthropod fragments and botanical elements were sampled from the body, from the coffin, and from the fabrics of his original clothes contained in the small box kept in the Saint’s reliquary.

### 2.2. Botanical Analyses

The various plant finds were divided by type and subsequently morphologically identified using a Leica Z16 APO 5.7-92x stereomicroscope. Reference texts used for the recognition of the samples are Renfrew [26] and the atlas by Bojnansky and Fargasovà [27].

### 2.3. Entomological Analyses

Fragments of insects and other arthropods were manually collected with sterile tweezers and paintbrushes from the basement of the coffin of Saint Davino and stored in vials. Adult insect fragments and puparia were mounted on entomological supports using a tiny amount of plasticine to fix the sample, then observed with a stereomicroscope Leica S9E (Leica, Wetzlar, Germany). The minimum number of individuals (MNI) was estimated with different approaches, depending on the morphological structure of the different taxa. For Diptera puparia, we considered as one specimen all the complete puparia or, if fragmented, only the fragments containing the posterior spiracles, which are very reliable diagnostic structures [11,28,29]. For adult Coleoptera, the number of elytra of the same side (left or right), the intact bodies, or abdomens with both elytra were used for the MNI calculation. The number of intact cocoons or cocoon remains, larger than half of their entire length, was used to estimate the number of Lepidoptera. For Diplopoda, only the head or the distal part of the body, easily recognizable, were considered. Identification was carried out at the lower level depending on the preservation of the fragments, using the specific identification keys and descriptions reported in Skidmore, [29], Smith, [30], Peacock [31], Giordani et al. [28], and Vienna [32]. In addition, fragments were compared with a reference collection by one of the authors (S. Vanin) made by modern and archaeological specimens to consider the taphonomic processes affecting the samples. Photographs were taken with a Nikon D5300 digital camera attached to a Leica Z16 APO stereoscope. Images were acquired using the StackShot TM multiple-focus imaging system and stacked in a single in-focus image using Stacker software ver. 1.04 and Helicon Focus 3D ver. 3.9.7W.

## 3. Results

### 3.1. Entomological Analysis

A total of 192 fragments belonging to a minimum number of 87 specimens were recovered from the Saint Davino coffin. The fragments belong to Coleoptera, Diptera, Lepidoptera, Hymenoptera (Insecta), Scorpiones (Arachnida), and Julida (Diplopoda). Notably, 98 (51%) fragments belong to the class Insecta, 91 (47%) to Diplopoda, and 3 (2%) to Arachnida. By considering the MNI, there were 84 insects, two diplopods, and a scorpion.

Among the insects, Diptera specimens were the most abundant, followed by Lepidoptera, Coleoptera, and Hymenoptera (Table 1).

**Figure 2:**
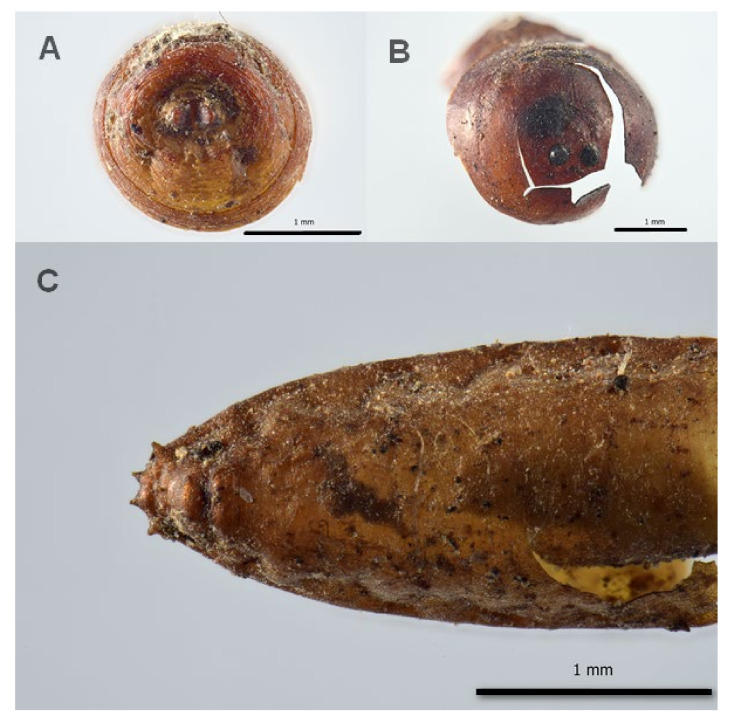
Diptera remains associated to the burial site of Saint Davino Armeno: (**A**) *Hydrotaea capensis*, puparia, posterior spiracles; (**B**) *Muscina* sp., puparia, posterior spiracles; (**C**) *Conicera* sp., puparia, posterior part.

Muscidae was the most represented family with 23 puparia belonging to *Hydrotaea capensis* (Wiedemann, 1818) and two puparia of a species in the genus *Muscina* (Robineau-Desvoidy, 1830). Phoridae was the second family with 13 puparia, which were identified as potentially belonging to the genus *Conicera* (Meigen, 1830) (Figure 2).

**Figure 3:**
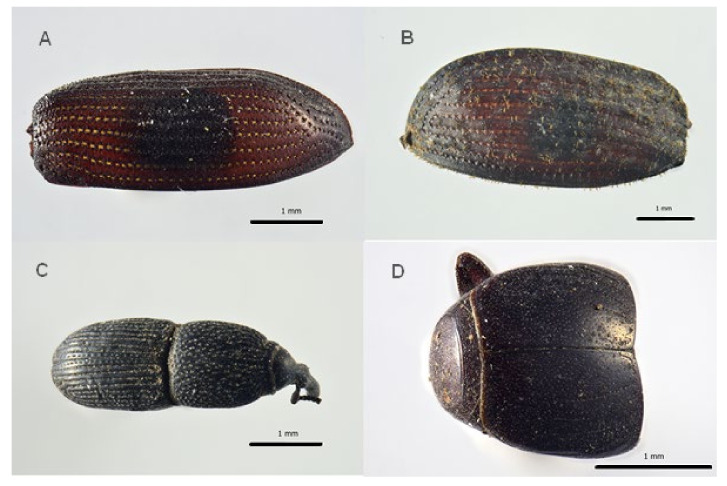
Coleoptera remains associated to the burial site of Saint Davino Armeno: (**A**) *Anobium punctatum* right elytra, (**B**) *Trox scaber*, left elytra, (**C**) *Sitophilus granarius* body, dorsal view, (**D**) *Gnathoncus* sp., abdomen dorsal view.

**Figure 4:**
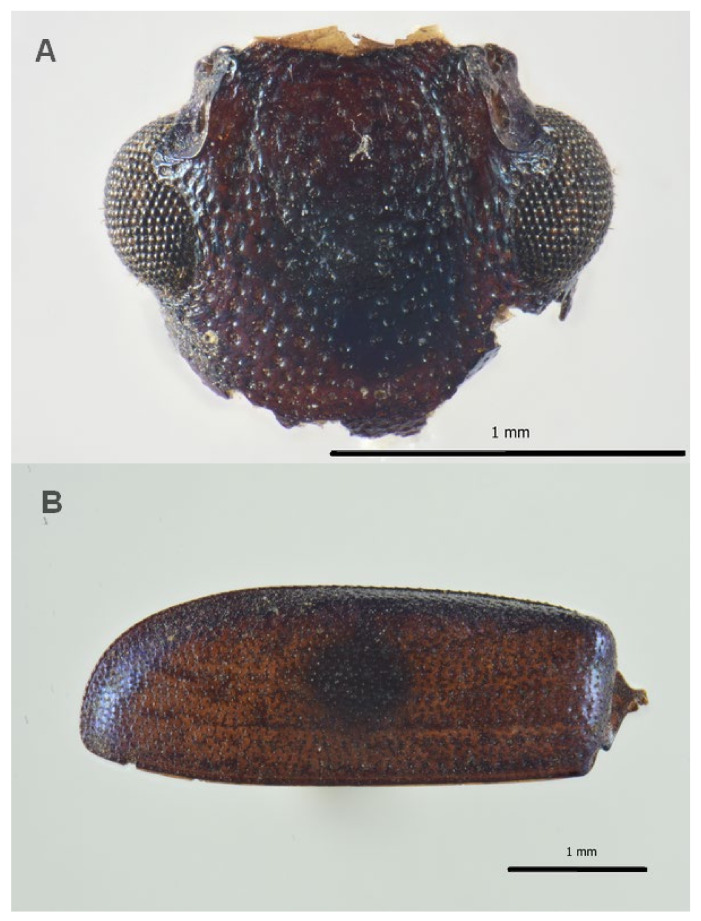
Coleoptera remains associated to the burial site of Saint Davino Armeno: (**A**) *Necrobia* sp., head, frontal view; (**B**) *Necrobia* sp., left elytra; note that the lack of bristles is due to the taphonomic process.

Among Coleoptera, Ptinidae were the most abundant with eight fragments (MNI 4) of the genus *Anobium* (De Geer, 1774) (Figure 3), which for the shape and distribution of the punctuation can be ascribed to *Anobium punctatum* (De Geer, 1774), followed by the family Cleridae with seven remains of the genus *Necrobia*, (Olivier, 1795) (MNI 4) (Figure 4), the family Trogidae, with five remains belonging to the genus *Trox scaber* (Linnaeus 1767) (MNI 4), and few fragments of the families Curculionidae (*Sitophilus granarius* (Linnaeus, 1758)) and Histeridae, genus *Gnathoncus* (Jacquelin du Val, 1857). A fragment of a mandible was not associated to any family and so was not counted as a specimen (Table 1).

**Figure 5:**
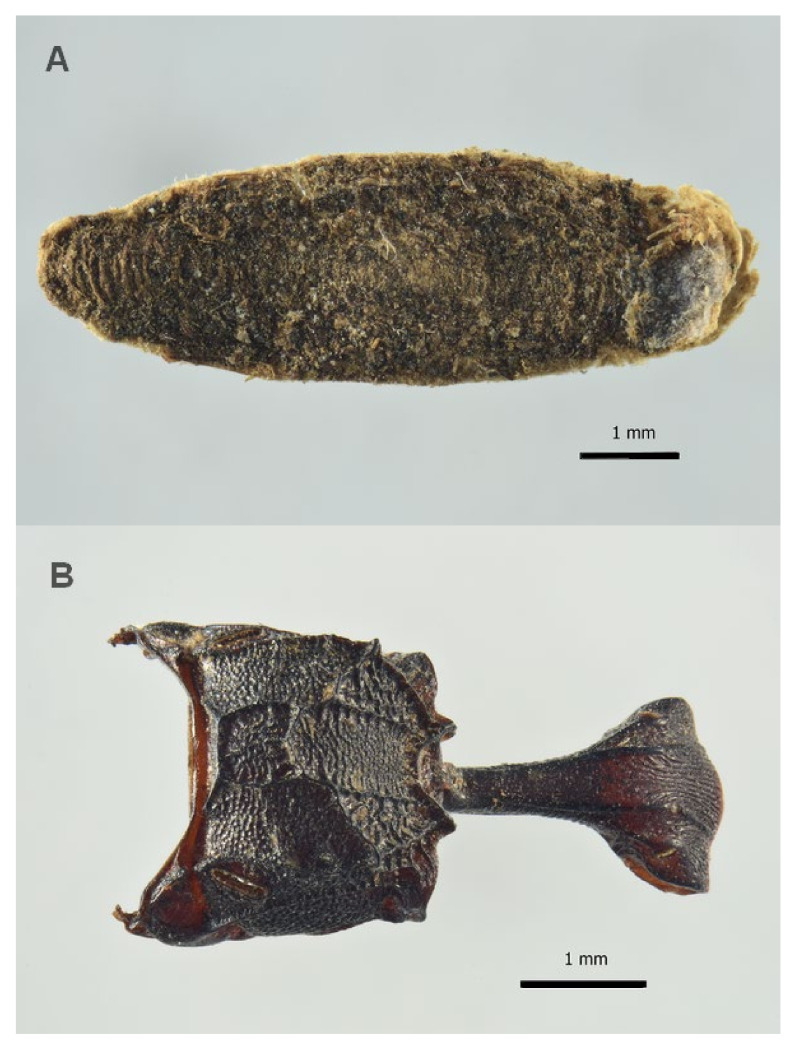
Lepidoptera and Hymenoptera remains associated to the burial site of Saint Davino Armeno: (**A**) Tineidae, cocoon; (**B**) Ichneumonidae, propodeum—petiole.

**Figure 6:**
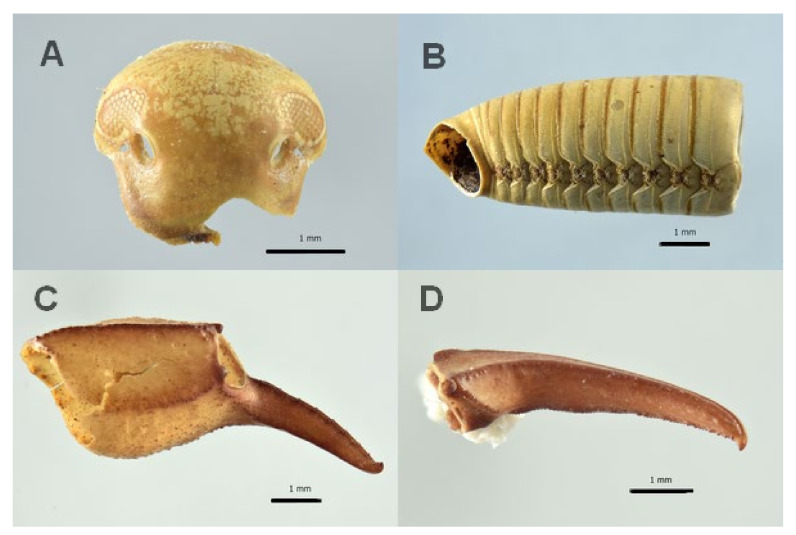
Julida and Scorpiones arthropod remains associated to the burial site of Saint Davino Armeno: (**A**) Julida head frontal view; (**B**) distal metamera; (**C**,**D**) Scorpiones, chela fragments.

Regarding Lepidoptera (Figure 5), only cocoons of specimens in the families Tineidae and Pyralidae were sampled (Figure 6A,B) (Table 1). A propodeum joined to the petiole and a mesopleuron of a specimen of Hymenoptera Ichneumonidae in the subfamily Ichneumoninae were sampled from the coffin (Figure 5). Numerous metamera of Julida were found, but only two terminal metamera were observed (Figure 6), allowing us to estimate a MNI of 2.

### 3.2. Botanical Analysis

The botanical remains identified in the Saint Davino coffin were from the following plant species: *Agropyron* cfr. *junceum* (L.) P. Beauv. (Gramineae) spikelet (1); *Bupleurum* cfr *rotundifolium* L. (Apiaceae) seeds (several); *Cuscuta* cfr. *europaea* L. (Convolvulaceae), seed (1); *Olea europaea* L. (Oleaceae), leaf fragments (several); *Phleum pratense* L. (Gramineae) kernels (several); *Quercus ilex* L. (Fagaceae) leaf fragments (several); *Setaria* cfr *pumila* (Poir.) Roem. et Schult. (Gramineae) kernel (1); *Vitis vinifera* L. (Vitaceae) seed (1). As a note, rodent droppings were also observed; these animals likely moved botanical remains.

## 4. Discussion

The information describing the burial of the body of Saint Davino is scarce and unclear. However, historical sources report that Saint Davino was initially buried in a grave pit in the cemetery located outside the church of Saint Michele in Foro (Figure 1), in the year 1050 [22,23]. This burial probably consisted of a simple wood coffin buried into the soil where people could walk, rest, and pray. A few years after the first burial, the corpse was transferred in a coffin of clay to the inside of the church and placed at the altar of Saint Luca along the north side of the church. In the 12th–13th centuries, the coffin of clay, with the body inside, was transferred to a larger marble sarcophagus. The body remained there for about four centuries, until the definitive exposure of the mummy to the veneration of the people under the high altar of the church started in 1592. Even if the historical reconstruction of the first events concerning the burial and the body of Saint Davino from the hagiographic tradition sources could be biased by legendary narrations, the entomological evidence can provide support for the historical information. All the insects were sampled from the last sarcophagus where the body of the Saint rested for about four centuries, beginning in 1646.

From an entomological point of view, almost all the arthropods belong to species which usually colonize corpses in the later colonization waves [30], but in concealed conditions, except for the species belonging to the genus *Muscina* (Diptera: Muscidae), which colonizes bodies during the first phases of decomposition [30]. This could suggest an initial colonization of the body when exposed at the time of the funeral. The total absence of Calliphoridae is interesting. Following the narrative sources, we could hypothesize the adoption of some procedures to prevent blowflies from reaching the body of the Saint, whose veneration was already affirmed when he was still alive. However, it could also be related to an initial exposure in an indoor place such as a sacristy or a church, even if close to buildings used by humans and livestock. Of note, rooms used as stables were certainly annexed to hospitals for pilgrims. A hospital for pilgrims, perhaps the same one that welcomed the pilgrim Saint Davino before his death, has been documented as being annexed to the church of Saint Michele in the 11th century [33]. This hypothesis is also consistent with the dominance of the species *H. capensis*, which often colonizes corpses that have been kept indoors under protected, confined environmental conditions, without exposure to open air [6,34].

The presence of Phoridae puparia is related to their ability to reach buried bodies up to one–two meters underground [35], a depth in the range of a wood coffin burial in a cemetery. The presence of elytra of *A. punctatum*, a xylophagous species [36] supports the hypothesis that a wood coffin was used in the first burial in an outdoor cemetery or, alternatively, this beetle species may have developed in the wood coffin in which Saint Davino has been exhibited since 1646. The former hypothesis is further supported by the finding of the thoracic and propodeum parts of Ichneumonidae wasps, a group of Hymenoptera strictly occurring in open spaces. Furthermore, the size of the specimens restricts the parasitisation activity of the species on large Lepidoptera larvae, with a mass body sufficiently abundant to allow the complete development of the hymenopteran parasitoid.

All the Lepidoptera associated with the body of Saint Davino belong to Tineidae or Pyralidae and the size of the collected cocoons were too small. The ichneumonid wasp host necessarily came from an external environment; we could hypothesize the presence of Lepidoptera larvae like those of Noctuidae or Pieridae, easily associable with an herb cover. *Sitophilus granaries* (Linnaeus, 1758), a stored product that beetle pest species often found in domestic environments [37], was likely developed on grain kernels, which were found inside the coffin.

Considering the botanical remains retrieved in the coffin, it can be argued that the Saint Davino remains stayed in a countryside characterized by mild temperatures and calcareous-clayey stony soil, with meadows composed of thermophilic grasses. The countryside was cultivated with vines and olive trees and surrounded by stony ground with evergreen sclerophylls (holm oaks). The plant remains were collected, together with dust and arthropod body parts, from a small wooden box containing the ancient robes of Saint Davino (various fabrics, mostly late medieval, used to dress the mummy up to the 16th–17th centuries). They are probably remains collected from the clay coffin during the moving of the body in the wooden shrine of 1646. They were collected because they were in contact with the body and therefore considered relics by contact. Both the wooden sarcophagus of 1646 containing the body and the wooden box with the clothes did not have holes, so if rodents (e.g., mice or rats) are responsible for the transport of botanical remains, they did it before 1646.

Even if we are not able to provide a precise dating to the plant remains and to their entry into the terracotta sarcophagus, it is very likely that they belong to the Middle Ages, and therefore return elements useful for the reconstruction of the urban landscape. The vine plant that miraculously grew on Davino’s burial mentioned in hagiographic sources can be seen as an element linked to the Christian Eucharistic symbolism, but it is interesting that the tradition remembers the presence of horticultural areas in the city centre, even near cemeteries. It is suggestive that a grape seed was found among the plant remains in Davino’s coffin. In the 11th century city, there must have been many cultivated areas with vegetable gardens and trees [38,39]. The olive tree, for example, of which we have several leaves among our findings, is testified in the medieval toponymy of Lucca: in the historic centre there was a place called “*All’Ulivo*” (i.e., “near the olive tree”), about 100 m east of the church of Saint Michele. Overall, the entomological analyses tell us that Saint Davino was first buried in the soil, probably in a wooden coffin, thus supporting the historical-hagiographic tradition according to which he was buried sub divo in the cemetery of Saint Michele. The preservation of the body as a natural mummy may in fact have been facilitated by burial in a coffin that prevented direct contact of the corpse with the earth. The botanical remains offer confirmation of a late medieval urban environment rich in horticultural areas and trees, giving us a landscape that is different from the current city.

As a final remark, it is noteworthy that the phenology of the species belonging to the genus *Muscina*, mainly active in late spring and early summer, provides additional support for the available information regarding the Saint’s death, which the hagiographic tradition places in June [40].

## Figures and Tables

**Table 1:** Number of fragments and minimum number of individuals (MNI) per taxon.

Order and Family	Species	Fragment(s)	No. of Fragments/MNI
Insecta			98/84
Diptera			36/35
Muscidae			23/22
	*Hydrotaea capensis*	Puparia	21/20
	*Muscina* sp.	Puparia	2/2
Phoridae	*Conicera* sp.	Puparia	13/13
Lepidoptera			35/33
Tineidae		Cocoon	29/27
Pyralidae		Cocoon	6/6
Coleoptera			25/15
Ptinidae	*Anobium punctatum*	Thorax/Elytrae	8/4
Cleridae	*Necrobia* sp.	Elytra/Head	7/4
Trogidae	*Trox scaber*	Elytra	5/4
Curculionidae	*Sitophilus granarius*	Body/Head/Abdomen	3/2
Histeridae	*Gnathoncus* sp.	Body	1/1
ND ^1^		Mandible	1/1
Hymenoptera			2/1
Ichneumonidae		Mesonotum Propodeum/Petiole	2/1
Julida		Metamera	91/2
Scorpiones		Chela	3/1
		Total	192/87

^1^ ND = not determined.

## Data Availability

Data are available from the corresponding author on reasonable request.
